# Asymptomatic Neurosyphilis Presenting With Bilateral Pulmonary Nodules: A Case Report

**DOI:** 10.1002/rcr2.70341

**Published:** 2025-09-08

**Authors:** Hiroshi Takahashi, Tadashi Ishida

**Affiliations:** ^1^ Department of Respiratory Medicine Kurashiki Central Hospital Okayama Japan

**Keywords:** asymptomatic neurosyphilis, multiple pulmonary nodules, *Treponema pallidum*

## Abstract

Globally, syphilis cases have been increasing and pulmonary lesions have been reported. However, asymptomatic neurosyphilis has not been documented. This condition is often unrecognised and presents with symptoms similar to those of other infectious and non‐infectious lung diseases. In syphilitic pneumonia, defined as a secondary form of syphilis, intramuscular injection of benzylpenicillin (24 million units) remains the gold standard for treatment, with a favourable prognosis. This report presents for the first time a case of asymptomatic neurosyphilis with multiple pulmonary nodules and buttock lipodystrophy. If rapid plasma reagin (RPR) antibody titres are high and RPR testing is difficult during follow‐up, cerebrospinal fluid (CSF) examination may be considered for further evaluation of asymptomatic neurosyphilis. This case showed a high expression of RPR (154 R.U.) and given the nature of the patient's work (long‐distance transportation), short‐term follow‐up was difficult. Therefore, CSF was performed to enable early diagnosis and treatment.

## Graphical abstract

This report presents for the first time, a case of asymptomatic neurosyphilis with multiple pulmonary nodules and buttock lipodystrophy. This case showed a high expression of RPR (154 R.U.) and given the nature of the patient's work (long‐distance transportation), short‐term follow‐up was difficult. Therefore, CSF was performed to enable early diagnosis and treatment.

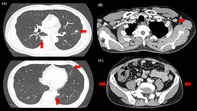

## 1 Introduction

Syphilis is a sexually transmitted infection (STI) caused by the spirochete *Treponema pallidum* . According to estimates by the World Health Organization (WHO), the number of new syphilis cases among adults aged 15–49 increased by approximately 900,000 from 2020 to 2022 with a global increase observed in various countries, including Japan [1, 2].

The clinical symptoms of syphilis are diverse, typically ranging from primary hard chancres to secondary mucocutaneous lesions, and further progressing to tertiary central nervous system disorders [3]. Pneumonia is a rare secondary symptom, with the first case report published in 1883 [4]. This condition is often unrecognised and presents with symptoms similar to those of other infectious and non‐infectious lung diseases [3, 4]. In syphilitic pneumonia, defined as the secondary form of syphilis, intramuscular injection of benzylpenicillin 24 million units remain the gold standard treatment, with a good prognosis following therapy [3]. However, delays in diagnosis and treatment may lead to complications, underscoring the importance of early detection and intervention.

Here, we report a rare case of asymptomatic neurosyphilis presenting with multiple nodules.

## 2 Case Report

**FIGURE 1:**
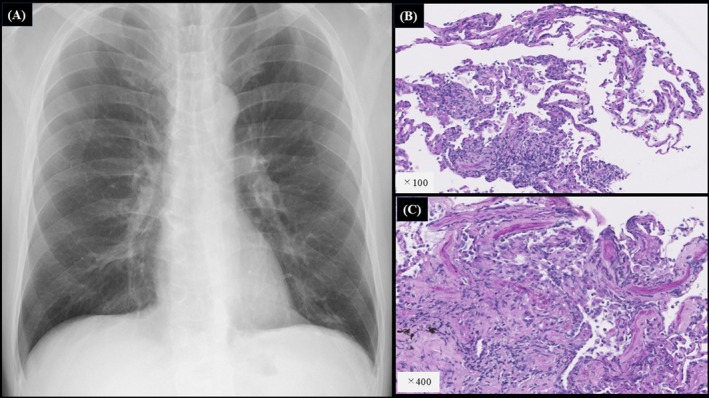
(A) Chest X‐rays showed multiple nodules in the bilateral lower lung fields. (B and C) The pathology of the right S^6^ lung on haematoxylin eosin staining showed numerous clusters of lymphocytes, plasma cells and numerous histiocytes within the alveolar septum.

**FIGURE 2:**
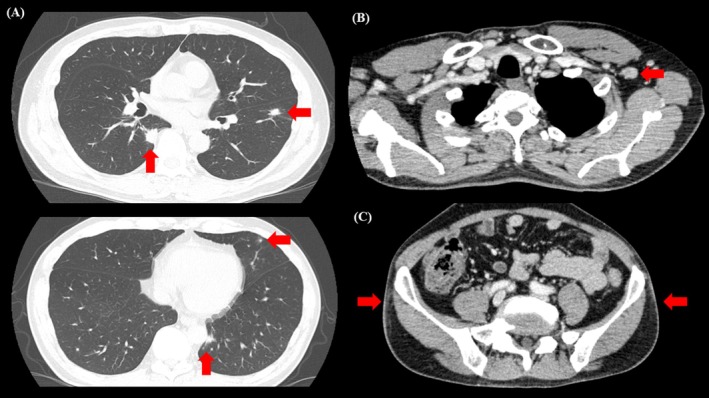
(A) The chest computed tomography (CT) showed predominantly multiple nodular and granular shadows just below the pleura in the bilateral lower lobes of the lungs. (B) The chest CT showed an enlarged left axillary lymph node. (C) The abdominal CT increased absorption values of subcutaneous adipose tissue on the outer bilateral iliac crest.

A 54‐year‐old man presented to the orthopaedic department with buttock pain lasting 1 month. Computed tomography (CT) showed incidental nodular shadows extending bilaterally to the lower lungs (Figures 1A and 2A), and he was referred to our department for suspected lung cancer. He also had a history of bronchial asthma and had previously smoked 20 cigarettes per day for 35 years. He had no spouse or sexual partner but had engaged in sexual contact with a commercial sex worker in his 30s.

His vital signs were as follows: body temperature: 37.1°C; pulse rate: 72 beats/min; blood pressure: 141/67 mmHg; respiratory rate: 12 breaths/min; SpO_2_ 97% (room air). Physical examination revealed no evident enlargement of the cervical or inguinal lymph nodes, and no subnoise was observed in his breath sounds. Small papules were predominantly observed in the medial aspect of both upper extremities. No obvious skin rashes were observed on the pubic region, trunk or buttocks.

**FIGURE 3:**
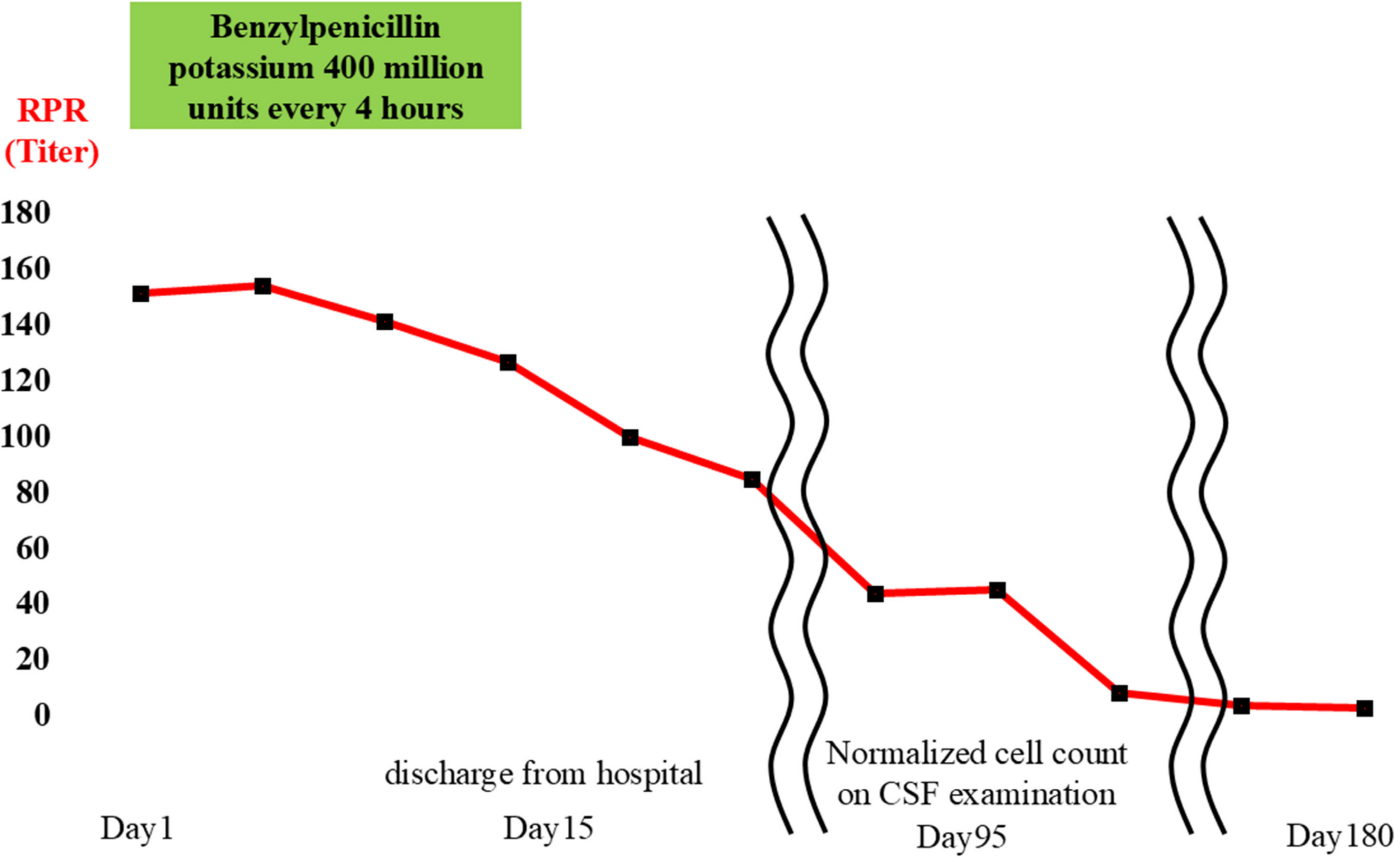
The clinical course of the case.

Serologic test results were as follows: C‐reactive protein, 4.66 mg/dL. There were no other abnormal findings on physiological or blood counts. Tumour markers (carcinoembryonic antigen, cytokeratin 19 fragment; neuron‐specific enolase; sialyl Lewis‐x antigen; pro‐gastrin‐releasing peptide); myeloperoxidase‐anti‐neutrophil cytoplasmic antibodies; proteinase‐3‐anti‐neutrophil cytoplasmic antibodies; and antinuclear antibodies were negative. The human immunodeficiency virus antibody; human T‐cell leukaemia virus type 1 antibody; interferon‐gamma release assay; hepatitis markers; *Mycobacterium avium‐intracellulare* complex antibody and β‐D glucan were also negative. The rapid plasma reagin (RPR) card test was 154.2 R.U.; and the TP antibody titre was 902.6 T.U. The cerebrospinal fluid test (CSF) results were as follows: the cell count was 34 cells/μL; mononuclear cells were 94%; polynuclear cells were 6% and protein was 30 mg/L. The RPR was less than 0.5 R.U., but fluorescent treponemal antibody absorption was positive. Tests for the cryptococcal antigen and film array 2.1 tests yielded negative results. Contrast‐enhanced magnetic resonance imaging of the head revealed no obvious intracranial lesions or cerebral aneurysms. On thoracoabdominal CT, in addition to diffuse nodular and granular shadows just below the pleura, the radiologist noted enlarged left axillary lymph nodes and increased absorption of subcutaneous adipose tissue on the outer bilateral iliac crests. (Figure 2A–C) Endobronchial ultrasonography with a guide sheath was performed on the biopsy of a nodule in the right S^6^. No obvious pathogens were noted, but there were numerous lymphocytes, plasma cells and histiocytes in the alveolar septum (Figure 1B,C). Treatment with 4 million units of potassium benzylpenicillin every 4 h was initiated for syphilis, and the RPR showed a rapid downward trend (Figure 3). Lung lesions due to syphilis were diagnosed when the following diagnostic criteria were met in a report by Coleman et al. [4]: (1) clinical course and physical findings typical of syphilis, (2) positive serological test for syphilis, (3) abnormal chest imaging findings with or without respiratory symptoms, (4) exclusion of causative diseases other than syphilis (vasculitis, other infectious diseases, malignant tumours, etc.) and (5) good response to treatment for syphilis. In this case, all the mentioned diagnostic criteria were met, and based on the results of the CSF examination, we diagnosed asymptomatic neurosyphilis with pulmonary involvement.

**FIGURE 4:**
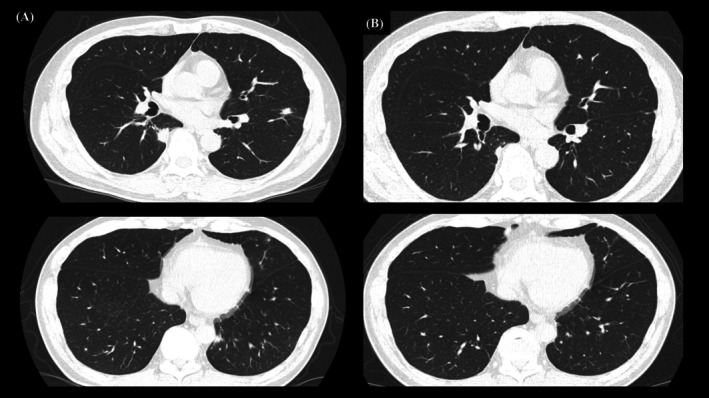
His nodular and granular shadows diffusely spreading to the bilateral lungs showed a tendency to disappear on the 180th day after treatment.

From the first day of admission, the patient was treated with 4 million units of potassium benzylpenicillin every 4 h for 2 weeks, as is appropriate for neurosyphilis. The patient was discharged from the hospital on the 15th day of admission as his buttock pain was relieved and RPR showed a decreasing trend. At 180 days after the start of treatment, the RPR was less than one‐third of the initial value, and the lung field nodules tended to disappear (Figure 4).

## 3 Discussion

This report presents the first reported case of asymptomatic neurosyphilis with multiple pulmonary nodules and lipodystrophy of the buttocks.

Lung lesions due to syphilis are reported to occur in 1%–12.5% of all cases of congenital syphilis and tertiary syphilis, and are rare even in cases of second‐stage syphilis [3], despite the appearance of some recent reports. However, to date, no reports of asymptomatic neurosyphilis patients presenting with multiple pulmonary nodules have been published. In a systematic literature review conducted on PubMed up to 1 June 2024, 43 cases of pneumonitis were identified, of which nodular lesions (32/43, 74.4%) and ground‐glass opacities (6/36, 16.7%) were observed most frequently on chest CT findings [3]. In this case, multiple pulmonary nodules were observed, consistent with the typical CT findings of pulmonary involvement in syphilis, which implies that syphilis should always be considered in the differential diagnosis of multiple nodules. According to a previous report, the characteristic histopathological findings on bronchoscopy of nodules caused by syphilis are the presence of granulomas and lymphoplasmacytic infiltrates [3], which are generally consistent with the pathological findings in this case. Although not performed for this case, PCR testing for syphilis in a transbronchial lung biopsy has also been reported to be useful for the diagnosis of syphilis and should be considered [3, 5].

In syphilis, lipodystrophy, such as erythema nodosum, has been reported to occur in tertiary syphilis but is considered relatively rare [6]. In this case, no obvious erythema nodosum could be noted from the skin findings; however, since the CT showed increased absorption of subcutaneous fatty tissue on the outer bilateral iliac crests, and pain in the same area, and since the subcutaneous fatty tissue disappeared after treatment for syphilis, we believe that the patient presented with lipodystrophy caused by syphilis.

*T. pallidum* has the potential to invade the central nervous system within a few days [7]. Asymptomatic neurosyphilis is present in 30% of early syphilis cases and 13.5% of late syphilis cases and is associated with a high risk of progression to symptomatic neurosyphilis [7]. Some cases of early asymptomatic neurosyphilis may progress to syphilitic meningitis, and in the late stages, may develop into meningovascular or chronic meningoencephalitis [7]. CSF is recommended for cases with neurological abnormalities according to the 2021 CDC guidelines. However, in cases where RPR antibody titres are high and RPR testing is difficult to follow‐up, CSF examination may be considered for further evaluation of asymptomatic neurosyphilis [7]. This case showed a high expression of RPR 154 R.U., and because of the nature of the patient's work (long‐distance transportation), short‐term follow‐up was difficult. Therefore, CSF was performed to enable the early diagnosis and treatment of asymptomatic neurosyphilis.

In conclusion, as in this case, depending on the recent epidemic history and endemic area, we believe that a close examination, including CSF examination, with syphilis in mind in the identification of bilateral multiple nodules, will lead to early diagnosis and early treatment.

## Author Contributions

Hiroshi Takahashi was responsible for drafting the manuscript; conception and design of the manuscript; and acquisition, analysis and interpretation of the data. Tadashi Ishida approved the final manuscript for publication.

## Consent

The authors declare that written informed consent was obtained for the publication of this manuscript and accompanying images using the form provided by the journal.

## Conflicts of Interest

The authors declare no conflicts of interest.

## Data Availability

Data sharing not applicable to this article as no datasets were generated or analysed during the current study.
